# Whole-Genome Sequencing of Human and Porcine *Escherichia coli* Isolates on a Commercial Pig Farm in South Africa

**DOI:** 10.3390/antibiotics13060543

**Published:** 2024-06-11

**Authors:** Wilhelmina Strasheim, Michelle Lowe, Anthony M. Smith, Eric M. C. Etter, Olga Perovic

**Affiliations:** 1 Centre for Healthcare-Associated Infections, Antimicrobial Resistance and Mycoses, National Institute for Communicable Diseases (NICD), a Division of the National Health Laboratory Service (NHLS), Johannesburg 2192, South Africa; 2 Department of Production Animal Studies, Faculty of Veterinary Science, University of Pretoria, Pretoria 0110, South Africa; 3 Department of Clinical Microbiology and Infectious Diseases, School of Pathology, Faculty of Health Sciences, University of Witwatersrand, Johannesburg 2193, South Africa; 4 Centre for Enteric Diseases, National Institute for Communicable Diseases (NICD), a Division of the National Health Laboratory Service (NHLS), Johannesburg 2192, South Africa; anthonys@nicd.ac.za; 5 Department of Medical Microbiology, School of Medicine, Faculty of Health Sciences, University of Pretoria, Pretoria 0084, South Africa; 6 CIRAD, UMR Animal, Santé, Territoires, Risque et Ecosystèmes (ASTRE), 97170 Petit-Bourg, France; 7 ASTRE, University of Montpellier, CIRAD, INRAE, 34398 Montpellier, France

**Keywords:** *Escherichia coli*, pigs, close human contacts, One Health, antibiotic resistance, whole-genome sequencing, virulence factors, core-genome MLST and hierarchical clustering, sequence type complex 10, South Africa

## Abstract

*Escherichia coli* is an indicator micro-organism in One Health antibiotic resistance surveillance programs. The purpose of the study was to describe and compare *E. coli* isolates obtained from pigs and human contacts from a commercial farm in South Africa using conventional methods and whole-genome sequencing (WGS). Porcine *E. coli* isolates were proportionally more resistant phenotypically and harbored a richer diversity of antibiotic resistance genes as compared to human *E. coli* isolates. Different pathovars, namely ExPEC (12.43%, 21/169), ETEC (4.14%, 7/169), EPEC (2.96%, 5/169), EAEC (2.96%, 5/169) and STEC (1.18%, 2/169), were detected at low frequencies. Sequence type complex (STc) 10 was the most prevalent (85.51%, 59/169) among human and porcine isolates. Six STcs (STc10, STc86, STc168, STc206, STc278 and STc469) were shared at the human–livestock interface according to multilocus sequence typing (MLST). Core-genome MLST and hierarchical clustering (HC) showed that human and porcine isolates were overall genetically diverse, but some clustering at HC2–HC200 was observed. In conclusion, even though the isolates shared a spatiotemporal relationship, there were still differences in the virulence potential, antibiotic resistance profiles and cgMLST and HC according to the source of isolation.

## 1. Introduction

Wild-type *Escherichia coli* is intrinsically susceptible to most antibiotics, but this bacterium has the ability to acquire a vast range of mobile genetic elements (i.e., virulence and antibiotic resistance genes) through horizontal gene transfer, which allows it to become pathogenic and/or resistant [1,2]. It is for this reason that *Escherichia coli* is used as an indicator micro-organism in One Health antibiotic resistance surveillance programs, as the antibiotic susceptibility profile of this bacterium demonstrates the existing antibiotic pressure inflicted on an environment [3,4].

Certain *E. coli* strains can cause a wide spectrum of diseases in both humans and animals [1]. The diseases in humans caused by *E. coli* can broadly be classified as (i) diarrheagenic or (ii) extraintestinal diseases (i.e., urinary tract infections (UTIs), peritonitis, bacteriaemia and meningitis) [5,6]. Diarrheagenic *E. coli* can further be classified into seven different pathovars, namely (i) enteropathogenic *E. coli* (EPEC), (ii) Shiga-toxin-producing *E. coli* (STEC), (iii) enterotoxigenic *E. coli* (ETEC), (iv) enteroaggregative *E. coli* (EAEC), (v) enteroinvasive *E. coli* (EIEC), (vi) diffusely adherent *E. coli* (DAEC) and adherent invasive *E. coli* (AIEC) [6,7].

Previously, the classification of *E. coli* pathovars relied on the presence or absence of group-specific virulence genes detected by a polymerase chain reaction (PCR) assay [8]. However, this method can only detect a limited number of virulence genes simultaneously [7]. This has changed in the era of whole-genome sequencing (WGS), as it is now possible to detect multiple virulence genes simultaneously without having any prior knowledge of the potential pathovar under investigation [6,7,8]. Whole-genome sequencing had shown that specific virulence factors, previously thought to be pathovar-specific, can be shared among different pathovars [6,7]. It is therefore important to define the epidemiological and clinical significance of different *E. coli* pathovars using WGS [6].

The primary ecological niche of *E. coli* is the gastro-intestinal tract of its vertebra host, where it normally exists as a commensal [6,9]. *Escherichia coli* may also be found in secondary habitats, such as water and soil sediments, and is used as an indicator of environmental fecal contamination [9,10]. The overlapping ecological niches of *E. coli* between humans, animals and the environment present the opportunity for the transmission of virulence and antibiotic-resistant genes, either (i) directly between humans and animals due to close proximity or (ii) indirectly through an intermediary vehicle, such as through the food chain or insect vectors [1,11]. Furthermore, virulence genes and antibiotic resistance genes are often located on mobile genetic elements, which can be transferred between groups of unrelated bacteria [2].

It is important to understand the transmission of antibiotic resistance and virulence factors, within a bacterium’s phylogenetic background, to develop interventions to reduce the burden of disease and antibiotic resistance [7,12]. However, the spread of antibiotic resistance at the human–livestock interface is somewhat controversial [12,13]. Earlier studies, using multilocus sequence typing (MLST) and pulsed-field gel electrophoresis, found the same sequence types (STs) and pulsotypes circulating in pigs and farmer workers [14]. However, MLST is based on seven different housekeeping genes and has a lower resolution than WGS [15]. Nowadays, WGS is considered as the gold standard, but there is still conflicting evidence of transmission at the human–livestock interface using a higher resolution technique [12,15].

A study conducted by Leekitcharoephon and colleagues (2021) investigated the genetic relatedness among 627 poultry, porcine and veal *E. coli* isolates from different farms in six European countries during 2014–2015 using WGS [16]. *Escherichia coli* isolated from the same farm, as well as different farms within the same country, showed some level of clonality but were genetically diverse between different animal species and different countries [16]. In addition, a study by Ludden and colleagues (2019) found limited evidence of clonality within a large collection of *E. coli* isolated from livestock and the food chain compared to isolates implicated in bloodstream infections in eastern England using WGS [17]. However, the isolates from the different sources were not isolated during the same time, and the majority of clinical isolates were healthcare-associated, which could be the potential reasons for the distinct lineages detected and limited evidence of transmission at the human–livestock interface in this setting [13,17].

The use of WGS combined with the appropriate study design can overcome these limitations and increase our understanding of complex transmission pathways [12]. Multiple studies on antibiotic resistance in *E. coli* have been conducted in South Africa, across various provinces, focusing on different production animals, humans, various stages in the food chain and the environment [18,19,20,21,22,23,24,25,26,27,28,29,30,31,32]. However, only a limited number of studies used WGS as a typing technique to investigate potential transmission events, and no studies were conducted on animal and human *E. coli* isolates that shared a direct spatiotemporal relationship at the farm level [18,33,34]. The study aimed to describe and compare *E. coli* isolates obtained from pigs and farm workers in close contact on a commercial pig farm in South Africa using conventional methods and WGS to investigate antibiotic resistance, virulence potential and phylogeny to identify potential transmission events.

## 2. Materials and Methods

### 2.1. Study Setting

Farm recruitment has been described previously [35]. The farm is situated in the North West province of South Africa and has a population of more than 1000 sows. The farm produces approximately 3000 metric tons of pork meat per year and has 25 production houses. Pigs are housed in four operational stages, namely (i) breeding, (ii) farrowing, (iii) weaning and (iv) growing.

### 2.2. Participant Recruitment, Sample Collection and Transportation

Sample collection was performed in December 2019. All farm employees (age ≥ 18 years) were invited to participate in the study after the completion of informed consent. Participants self-collected a dual-tip rectal swab (BD BBL CultureSwab EZ Swab, Franklin Lakes, NJ, USA) and completed a study questionnaire to collect the following variables: (i) age, (ii) sex and (iii) routine farm duties (i.e., animal handler, market transportation, routine maintenance and housekeeping or other). Four to five fresh, undistributed pig fecal droppings, weighing approximately 10 g, were collected aseptically from randomly selected pens per production house for all production stages. All samples were transported by road and processed within 48 h of collection at the Centre for Healthcare-Associated Infections, Antimicrobial Resistance and Mycoses (CHARM), National Institute for Communicable Diseases (NICD), a division of the National Health Laboratory Service (NHLS).

### 2.3. Isolation, Identification and Antibiotic Susceptibility Testing (AST) of E. coli

A primary inoculum was performed with the self-collected rectal swab on a MacConkey agar plate (Diagnostic Media Products, Johannesburg, South Africa). The inoculum was streaked for single colonies and incubated aerobically at 35 °C (±2 °C) for 18–24 h. Ten grams (10 g) of the pig fecal droppings was added to 90 mL of buffered peptone water (BPW) (Oxoid, Thermo Fisher Microbiology, Basingstoke, Hampshire, United Kingdom) and vortexed to homogenize the sample. The inoculated BPW was incubated at 35 °C (±2 °C) for 18–24 h. The next day, 10 µL of the BPW was plated onto MacConkey agar (Diagnostic Media Products, Johannesburg, South Africa) and incubated under the same conditions.

The identity of all (both human rectal swabs and pig fecal droppings) presumptive *E. coli* colonies was confirmed with matrix-assisted laser desorption/ionization time of flight mass spectrometry (MALDI-TOF MS) (Bruker Daltonics, Billerica, MA, USA). The MicroScan WalkAway plus (Beckman Coulter, Brea, CA, USA) system was used for antibiotic susceptibility testing using the NM44 panel. The minimum inhibitory concentration (MIC) of colistin was determined with the Sensititre system using custom-made plates (FRCOL) (Thermo Fisher Scientific, Waltham, MA, USA). The MIC for ciprofloxacin was determined using a gradient diffusion method (Etest^®^, BioMerieux SA, Marcy l’Etoile, France) to detect low-level ciprofloxacin resistance (from 0.06 µg/mL to 0.5 µg/mL). The gradient diffusion method (Etest^®^, BioMerieux SA, Marcy l’Etoile, France) was also used to detect the MIC of streptomycin, as this antibiotic was not included in the commercial NM44 panel. The Clinical and Laboratory Standards Institute (CLSI) (M100, 2020) guidelines were used for the interpretation of MIC for all antibiotics, except for streptomycin, colistin and tigecycline [36]. The epidemiological cut-off (ECOFF) value of ≥32 µg/mL, as established by the US National Antimicrobial Resistance Monitoring System (NARMS), was used for streptomycin, whereas the European Committee on Antimicrobial Susceptibility Testing (EUCAST) guidelines (v.13.0, 2023) were used for colistin and tigecycline [37,38].

### 2.4. Total Genomic DNA Extractions and Whole-Genome Sequencing (WGS)

A purity plate prepared during AST was used to inoculate Brain Heart Infusion (BHI) broth (Diagnostic Media Products, Johannesburg, South Africa) for each *E. coli* isolate. The BHI broth was incubated at 35 °C (±2 °C) for 18 h to serve as the starting material for DNA extractions. Total genomic DNA extractions were performed using the QIAamp DNA Minikit (Qiagen, Hilden, Germany) according to the manufacturer’s instructions. The DNA extracts were submitted to the Sequencing Core Facility (SCF), NICD, for WGS. The Nextera DNA Flex library prep kit (Illumina, San Diego, CA, USA) was used for library preparation, with the inclusion of a gBlock Gene Fragment (Integrated DNA technologies, Coralville, IA, USA) as a quality control measure. The Illumina NextSeq 550 instrument (Illumina, USA) was used for sequencing at 100× coverage, using 2 × 150 base pairs (bp) paired-end sequencing for each flow cell, with the addition of a PhiX v.3 (Illumina, San Diego, CA, USA) to serve as a cluster generation and sequencing control.

### 2.5. Bioinformatics Analysis

The JEKESA pipeline (v 1.0) was used in part for the processing of sequencing reads [39]. In short, quality control was performed using FastQC (v.0.11.9) (Available online: https://www.bioinformatics.babraham.ac.uk/projects/fastqc/, accessed on 23 October 2021), and paired-end reads were trimmed with Trim Galore! (v.0.6.7) (Available online: https://github.com/FelixKrueger/TrimGalore, accessed on 5 August 2021), with the Q-score and read length parameters set at >30 bp and >50 bp, respectively. The presence of contaminating sequences other than *E. coli* was assessed with Kraken 2 [40]. The assembly was performed using SPAdes (v.3.14.1) and polished in shovill (v.1.1.0) (Available online: https://github.com/tseemann/shovill, accessed on 5 August 2021) [41]. The final read assembly was assessed using QUAST [42].

Annotation of the assembled genomes was performed using VirulenceFinder 2.0 and ResFinder 4.1 hosted on the Centre for Genomic Epidemiology server, with the pre-set parameters [43,44,45,46,47]. Enteroaggregative *E. coli* was defined based on the definition proposed by Boison and colleagues (2020), which states that an *E. coli* strain should contain AggR (*agg*R together with its Agg-R-activator regulator protein encoded by the aar gene) and complete aggregative adherence fimbriae (AAF) (I–V) or CS22 colonization factor gene cluster [48]. Enteropathogenic *E. coli* was defined based on the presence of the locus of enterocyte effacement (LEE) pathogenicity island, as well as the presence of non-LEE effectors [49]. Enterotoxigenic *E. coli* isolates were defined based on the virulence factors listed by Duan et al., 2012 and Pakbin et al., 2021 [50,51]. Extraintestinal pathogenic *E. coli* isolates were defined based on the presence of Group II or Group III capsules, which protect ExPEC from phagocytosis and complement-mediated killing by the host’s immune system [52,53]. An *E. coli* isolate had to harbor at least two of the above-mentioned virulence factors simultaneously to be classified as ExPEC. Shiga-toxin-producing *E. coli* was defined as any isolate that harbored the *stx* genes.

Raw sequencing data (FastQ files for paired-end reads) were uploaded to the EnteroBase platform (Available online: https://enterobase.warwick.ac.uk/species/index/ecoli, accessed on 23 October 2021), where various tools were used to investigate phylogeny, namely (i) MLST, (ii) Clermont phylogroups and (iii) core-genome MLST (cgMLST) and hierarchical clustering (HC) among human and porcine *E. coli* isolates [54,55,56,57]. The genomic relationships were visualized using GrapeTree with the MSTree V2 algorithm based on the cgMLST V1 + HierCC V1 scheme [58]. All sequencing reads were deposited in the National Centre for Biotechnology Information (NCBI)’s GenBank under BioProject number: PRJNA994298.

### 2.6. Statistical Analysis

Data were captured in Microsoft Excel 2016. Data cleaning and analysis were performed in R (v4.0.2) using the janitor, dplyr and rstatix packages [59,60,61]. The categorical variables were summarized as numbers and percentages. Pearson’s chi-squared test with Yates’ continuity correction was used to compare the differences between human and porcine *E. coli* isolates. A *p*-value of 0.05 was considered significant.

## 3. Results

### 3.1. Participant Demographics and the Isolation Rate of E. coli

Sixty-four (64) farm workers were recruited, of whom the majority were male (76.56%, 49/64). The participants were on average 40 years old (range: 22 years–67 years). The routine farm duties were unknown for six participants (9.38%, 6/64). The remaining participants were involved in various duties, which included animal handling (56.90%, 33/58), maintenance and housekeeping (31.03%, 18/58), transportation of pigs to the abattoir (8.62%, 5/58) and working in the feeding mill (3.45%, 2/58). Each participant (*n* = 64) self-collected a rectal swab, and *E. coli* was isolated from 78.13% (50/64) of the swabs. Different colony morphologies of *E. coli* isolates were observed from the same rectal swab in thirteen instances, and two isolates were therefore processed. Thus, a total of 63 human *E. coli* isolates obtained from 50 rectal swabs underwent further testing.

A total of 113 pig fecal droppings were collected from 23 production houses. *Escherichia coli* was not isolated from every pig fecal dropping (detection rate: 88.5%, 100/113), but it was isolated from every production house (100%, 23/23) across all production phases (100%, 4/4). *Escherichia coli* isolates with different colony morphologies were also observed in five pig fecal droppings (two different colony morphologies in four droppings and three different colony morphologies in a single dropping). Thus, a total of 106 porcine *E. coli* isolates obtained from 100 pig fecal droppings underwent further testing.

### 3.2. Phenotypic Antibiotic Resistance Testing

The phenotypic resistance rates for human and porcine *E. coli* isolates are shown in Table 1. Overall, high levels of resistance were observed for ampicillin (71.60%, 121/169) and tetracycline (71.01%, 120/170), whereas moderate levels of resistance were observed for trimethoprim-sulfamethoxazole (32.54%, 55/169), streptomycin (26.04%, 44/169), chloramphenicol (24.26%, 41/169) and ciprofloxacin (10.65%, 18/169). Low levels of resistance toward cephems (0.59%, 1/169), monobactams (0.59%, 1/169), fosfomycin (1.18%, 2/169) and gentamicin (4.14%, 7/169) were observed. Colistin and carbapenem resistance was not detected. A single porcine *E. coli* isolate (P74) had a MIC breakpoint of >2 µg/mL for tigecycline. Porcine *E. coli* isolates were more resistant than human isolates toward ampicillin, piperacillin, ampicillin-sulbactam, tetracycline, ciprofloxacin and chloramphenicol (*p* < 0.05).

### 3.3. Genotypic Antibiotic Resistance Profiles

The genotypic resistance rates, as determined by ResFinder (v4.1) for human and porcine *E. coli* isolates, are shown in Table 2.

No acquired antibiotic resistance genes (i.e., no hits found) were detected for fosfomycin, fusidic acid, nitroimidazoles, oxazolidinones and glycopeptides. A single porcine isolate (P42) harbored a point mutation (V161G) in the *pmr*B gene but was not phenotypically resistant to colistin. The antibiotic resistance genes for aminoglycosides, ß-lactams, quinolones and tetracyclines are further summarized below.

#### 3.3.1. Aminoglycoside-Modifying Enzymes (AMEs)

All three classes of aminoglycoside-modifying enzymes (AMEs), namely acetyltransferases (ACC), nucleotidyltransferases (ANT) and phosphotransferases (APH), were detected in human and porcine *E. coli* isolates. In addition, multiple combinations of APHs, which encode resistance toward streptomycin and kanamycin, were detected. A richer diversity of APHs and ANTs was detected in porcine *E. coli* isolates compared to human *E. coli* isolates (*p* < 0.05).

#### 3.3.2. ß-Lactam Resistance Genes

Beta-lactam resistance was predominantly mediated by different variants of the *bla*_TEM_ gene in both human and porcine *E. coli* isolates (64.50%, 109/169), with the exception of the detection of *bla*_OXA-1_ in combination with *bla*_TEM-1B_ in 5.66% (6/106) of the porcine *E. coli* isolates. Sixty-eight percent (68.25%, 43/63) of human *E. coli* isolates did not harbor a ß-lactam antibiotic resistance gene, whereas the majority of the porcine *E. coli* isolates harbored a resistance gene associated with ampicillin resistance (89.62%, 95/106) (*p* < 0.05).

#### 3.3.3. Quinolone Resistance Genes

Quinolone resistance was mediated by chromosomal point mutations, as well as antibiotic resistance determinants residing on plasmids. One was more likely to detect both the S83L point mutation in DNA gyrase (*gyr*A) gene and OqxAB efflux pump in porcine than in human *E. coli* isolates (*p* < 0.05). Human *E. coli* isolates were more likely not to harbor any plasmid-mediated antibiotic-resistant determinants for fluoroquinolones than porcine *E. coli* isolates (*p* < 0.05).

#### 3.3.4. Tetracycline Resistance Genes

A total of 71.43% (45/63) of human *E. coli* isolates did not harbor a tetracycline resistance gene, in contrast to the 4.72% (5/106) of porcine *E. coli* isolates without a tetracycline resistance determinant (*p* < 0.05). Overall, tetracycline resistance was mediated by three genes, namely *tet*A, *tet*B and *tet*M, of which *tet*A occurred with the highest frequency (36.69%, 62/169), followed by *tet*B (34.32%, 58/169). The *tet*M gene was not detected in human isolates and was always detected in combination with either *tet*A (4.72%, 5/106) or *tet*B (11.32%, 12/106) in porcine isolates. A single porcine *E. coli* isolate harbored the *tet*A and *tet*B genes simultaneously.

### 3.4. Virulence Potential

Overall, 103 different types of virulence genes were detected. Thirteen virulence genes were only detected in porcine *E. coli* isolates (12.62%, 13/103), whereas forty-seven different virulence genes (45.63%, 47/103) were only detected in human *E. coli* isolates (*p* < 0.05). All isolates (100%, 169/169) harbored the tellurite resistance (*ter*C) gene. The occurrence of virulence genes was further interpreted based on the virulence gene combinations that grouped into gene clusters, pathogenicity islands and pathotypes (Table 3).

The following pathovars were detected at low frequencies and is described in more detail below: (i) extraintestinal *E. coli* (ExPEC) (12.43%, 21/169), (ii) ETEC (4.14%, 7/169), (iii) EAEC (2.96%, 5/169), (iv) EPEC (2.96%, 5/169) and (v) STEC (1.18%, 2/169). Overlaps between the ETEC and ExPEC pathovars in three porcine isolates, as well as in EAEC and ExPEC pathovars in two human isolates, were observed. The majority of ETEC (85.71%, 6/7), EPEC (80.00%, 4/5) and all STEC (100.00%, 2/2) pathovars were isolated from pigs, whereas all EAEC (100.00%, 5/5) pathovars and the majority of ExPEC (80.95%, 17/21) pathovars were isolated from healthy human volunteers.

#### 3.4.1. Enteroaggregative *E. coli* (EAEC)

The complete molecular gene signatures associated with EAEC were only detected in three human *E. coli* isolates (H24-2, H31 and H59-2) and not in any of the porcine isolates. These three human isolates also harbored the *aai*C gene cluster with the putative proteins ORF3 and ORF4, as well as the dispersin (*aap*) and the dispersin transporter protein (*aat*A) genes simultaneously. Two human *E. coli* isolates (H21 and H54) harbored a complete AFA-III gene cluster (H21: *afa*ABCDE; and H54-1: *afa*ABCDE8), but the AggR transcriptional activator was not detected. Various other genes (*sat* and *sig*A) previously reported to be associated with EAEC were also mostly detected in the human *E. coli* isolates (*p* < 0.05).

#### 3.4.2. Enteropathogenic *E. coli* (EPEC)

A total of five *E. coli* isolates were classified as EPEC, of which a single isolate originated from a human (H27-2), and four isolates (P6-2, P75, P105 and P107) originated from pigs. All porcine EPEC isolates were detected in different production houses but originated from two production stages, namely the weaning phase (P6-2 and P75) and the growing phase (P105 and P107).

#### 3.4.3. Enterotoxigenic *E. coli* (ETEC)

The virulence factors associated with ETEC detected in this study included *ast*A, *ltc*A, *mbc*A, F18 fimbriae (*fed*AF) and STb toxin (*stb*). An *E. coli* isolate was defined as ETEC in this study if it harbored at least two of the above-mentioned virulence factors simultaneously. A total of six porcine *E. coli* isolates (P10-2, P91, P102, P97, P98-1 and P35) and a single human *E. coli* isolate (H29) harbored the following gene combinations: (i) *ast*A-*stb* (P10-2, P102, P97 and P98-1), (ii) *ast*A-*mcb*A (P91), (iii) *ast*A-*fed*AF (P35) and (iv) *ast*A-*ltc*A (H29).

#### 3.4.4. Extraintestinal Pathogenic *E. coli* (ExPEC)

Human ExPEC harbored different Group II capsules, namely K1, K5, K23 and K52. Four pig isolates (P60, P97, P98-1 and P102) harbored the *kps*MII capsule, whereas two porcine isolates (P68 and P113) only harbored the *kps*E transporter protein. Three porcine isolates (P97, P98-1 and P102) also simultaneously harbored genes associated with ETEC, whereas two human isolates (H21 and H54-1) also simultaneously harbored genes associated with EAEC.

#### 3.4.5. Shiga-Toxin-Producing *E. coli* (STEC)

Two porcine isolates [P92 (ST162, phylogroup B1, O8:H28) and P109 (ST23, phylogroup B1, O8:H9)] from the growing production phase were classified as STEC. These isolates harbored the Shiga toxin type 2 (Stx2) (*stx*2AB) and were detected in different production houses (House 19 and House 22).

#### 3.4.6. Other Virulence Genes Detected That Are Not Associated with a Specific Pathovar

Multiple other virulence genes that overlap between the different pathovars or that are not associated with a specific pathovar were also detected. These virulence factors were further grouped according to function and are available in the Supplementary Material (Table S1). Multiple virulence factors encoding bacteriocins were detected. In addition, genes associated with iron acquisition, colonization and toxins (*hly*A and *tox*B) were also detected.

### 3.5. Phylogeny

#### 3.5.1. Clermont Phylogroups

Overall, most *E. coli* isolates were assigned to Clermont phylogroup A (63.31%, 107/169), followed by phylogroup B1 (23.08%, 39/169), whereas phylogroups B2, C, D, E and F were detected at low frequencies (Table 4).

Clermont phylogroup A was more frequently detected in porcine *E. coli* isolates than in human isolates (*p* < 0.05), whereas Clermont phylogroup D was more frequently detected in human isolates than in porcine isolates (*p* < 0.05).

#### 3.5.2. Multilocus Sequence Typing—Sequence Type Complexes

The sequence type complex (STc) could not be assigned for 36.09% (61/169) of *E. coli* isolates. Among the remaining isolates, 18 different STcs were detected overall, of which 4 STcs (STc23, STc32, STc165 and STc467) were only detected in porcine isolates, and 8 STcs (STc69, STc95, STc101, STc155, STc156, STc394, STc399 and STc522) were only detected in human isolates (Table 5).

Six different STcs (STc10, STc86, STc168, STc206, STc278 and STc469) were shared between human and porcine isolates. There was no statistically significant difference in the distribution between STs among human and porcine *E. coli* isolates, except for STc 69, which was more frequently detected in human *E. coli* isolates (*p* < 0.05).

The composition of each STc with the different STs is listed in Supplementary Table S2. Sequence type 10 (22.49%, 38/169), followed by ST542 (5.92%, 10/169), were detected with the highest frequency. Different STs were detected for human and porcine isolates within the same STc (i.e., STc168, STc206 and STc278). For example, within STc168, ST93 was only detected in porcine *E. coli* isolates, whereas ST484 was detected in a single human *E. coli* isolate.

#### 3.5.3. Core-Genome Multilocus Sequence Typing (cgMLST) and Hierarchical Clustering (HC)

**Figure 1:**
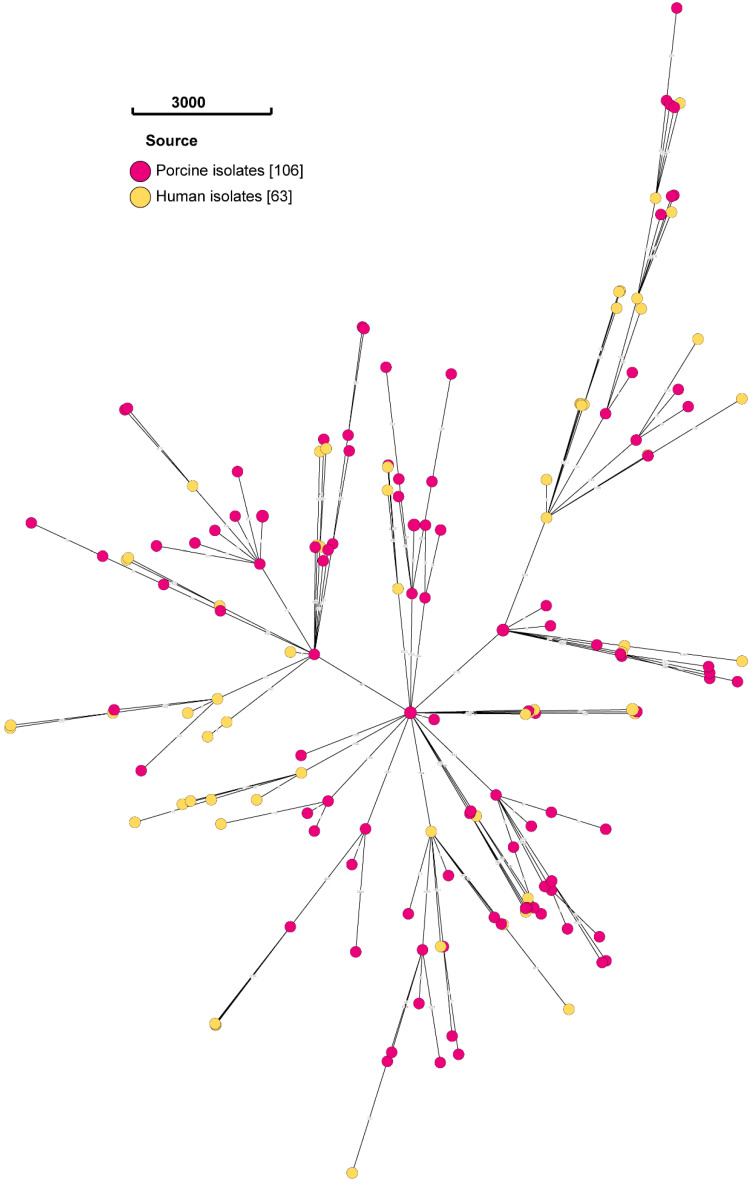
Minimum spanning tree based on the core-genome MLST and hierarchical clustering (HC) of human (yellow dots) and porcine (pink dots) *E. coli* isolates on EnteroBase, visualized using GrapeTree.

The overall phylogeny of *E. coli* isolates is shown in the minimum spanning tree (Figure 1). Five clusters of genetically indistinguishable isolates were detected at HC level 0 (HC0), namely HC0:ST171955, HC0:ST173766, HC0:ST173767, HC0:ST173811 and HC0:ST173815. Each cluster consisted of two isolates. HC0:171955 was only detected in humans, whereas HC0:ST173767 originated from the same pig but was morphologically distinct. The three other STs (i.e., HC0:ST173766, HC0:ST173811 and HC0:ST173815) originated from the same production stage (i.e., weaning), but each cluster of weaner *E. coli* isolates originated from a different production house (House 2, House 12 and House 16).

**Figure 2:**
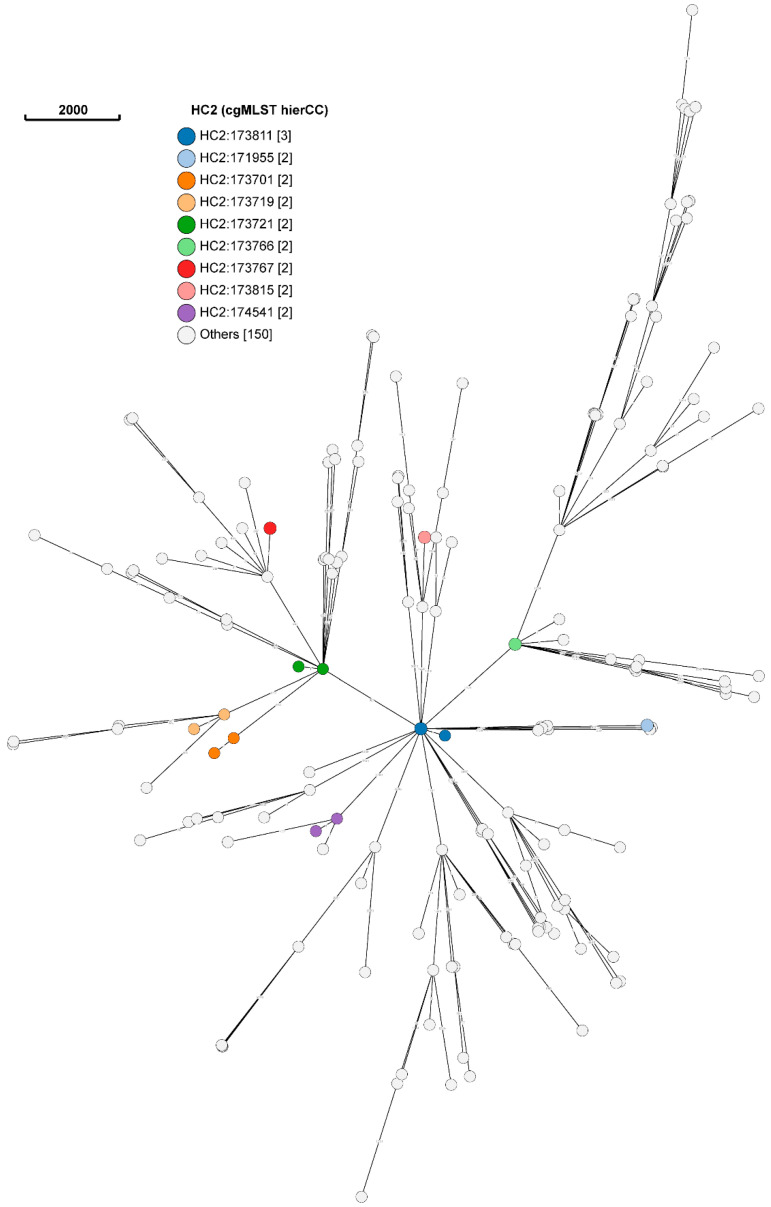
Minimum spanning tree of human and porcine *E. coli* isolates at hierarchical level 2 on EnteroBase, visualized using GrapeTree.

A total of nine clusters were detected at HC level 2 (HC2), namely HC2:173881, HC2:171955, HC2:173701, HC2:173719, HC2: 173721, HC2:173766, HC2:173767, HC2:173815 and HC2:174541 (Figure 2).

Each cluster consisted of two isolates, with the exception of HC2:173811, which consisted of three isolates. Three clusters (i.e., HC2:171955, HC2:173701 and HC2:173719) consisted of only human isolates, whereas five clusters (i.e., HC2:173766, HC2:17367, HC2:173811, HC2:173815 and HC2:174541) consisted of only porcine isolates. Hierarchical cluster 2:173721 (HC2:173721) consisted of a human (H47) and porcine (P101) *E. coli* isolate, which provides some evidence for the transmission and subsequent spread of antibiotic resistance genes between humans and animals.

Additional evidence of the clustering of human and porcine isolates was revealed at HC10, with the detection of HC10:173841. This HC consisted of a single human *E. coli* isolate (H15-1) isolated from a female animal handler working in the weaning production stage and a porcine *E. coli* isolate (P52) isolated from a dry sow at the same site (i.e., Site B) on the farm. A total of three clusters of human and porcine isolates were detected at HC level 200 [i.e., HC200:3556, consisting of human *E. coli* (H27-1) and porcine *E. coli* (P4) isolate; HC200:41988, previously HC10:173841, but with an additional porcine *E. coli* P53 isolate; and HC200:32433, previously HC2:173721], whereas a total of seven clusters of human and porcine *E. coli* isolates were detected at HC level 400 (i.e., HC400:13, HC400:37, HC400:82, HC400:4483, HC400:4993, HC400:5951 and HC400:31574).

## 4. Discussion

*Escherichia coli* serves as an indicator micro-organism in antibiotic resistance surveillance programs [4]. Whole-genome sequencing can concurrently provide information on an isolate’s antibiotic resistance profile, its virulence potential and its genetic relationship with other *E. coli* isolates [62]. This can provide insights into the spread of antibiotic resistance genes among the One Health continuum [62]. The study found that porcine *E. coli* isolates were more resistant and harbored a richer diversity of antibiotic resistance genes than *E. coli* isolated from close human contacts on the same farm, specifically for penicillins (i.e., *bla*_TEM-1_), tetracyclines (i.e., *tet*A and *tet*B), ciprofloxacin (i.e., S83L point mutation in *gyr*A and OqxAB efflux pump) and phenicols (*p* < 0.05). On the other hand, a richer diversity of virulence genes associated with the specific pathotypes was detected in human *E. coli* isolates (*p* < 0.05). Although the same STcs were circulating in both pigs and close human contacts, only a single set of human and porcine *E. coli* isolates showed clonality at HC2, which is an indication of a recent transmission event.

The high rates of tetracycline and ampicillin resistance described in this study are in line with global antibiotic resistance rates described in various food-producing animals [63]. The antibiotic resistance rates in this study were also similar to a study conducted in an intensive pig production system in KwaZulu Natal, South Africa, where the highest rate of resistance detected was toward tetracyclines, and the lowest rate of resistance detected was toward carbapenems [64]. It is well established that tetracycline resistance in the South African agricultural setting is high, which may be a reflection of the antibiotic practices in South Africa [34,65,66]. Tetracycline can be purchased over the counter and used at the farmer’s own discretion [67]. A previous study by the authors (2022) showed that tetracyclines were the antibiotic class used in the highest quantity (i.e., 453.65 ± 35.49 kg or 135.16 mg/kg ± 3.31 mg/kg) on the same farm, which will explain why tetracycline resistance was high (95.28%, 101/113) in porcine isolates and was predominantly mediated by the *tet*A and *tet*B genes [35].

Tetracycline and ampicillin resistance determinants are often harbored on the same plasmid, which may explain why ampicillin and tetracycline resistance was equivalent in the porcine *E. coli* isolates [68]. Ampicillin resistance was predominantly mediated by the *bla*_TEM-1_ gene, which has previously been reported in non-O157 *E. coli* isolates in cattle farming from the same province [34]. The level of ampicillin resistance in this study was also higher compared to a study performed on swine farms in southern Brazil [11]. Brisola and colleagues (2019) found that the level of ampicillin resistance was 11.76% (8/103) compared to 94.34% (100/113) found in the porcine isolates in this study [11]. The high levels of ampicillin resistance are again potentially a reflection of the antibiotic practices on the farm, where ampicillin is used as metaphylaxis for post-weaning diarrhea [35].

Multiple porcine *E. coli* isolates harbored the multidrug efflux pump, OqxAB, which confers resistance to a veterinary growth promotor, olaquindox, but also to chloramphenicol, tigecycline, nitrofurantoin, fluoroquinolones, detergents and disinfectants [69,70]. This efflux pump is located on a plasmid and flanked by two insertion sequence (*IS*26) elements, which means it can easily be acquired by other bacteria, such as *Klebsiella pneumoniae*, through horizontal gene transfer [70,71]. Strasheim and colleagues (2022) previously reported that olaquindox was the antibiotic class used in the second highest quantity (i.e., 258.33 ± 8.04 kg or 77.07 mg/kg ± 3.93 mg/kg) on the farm [35]. Antibiotic growth promotors are not banned in South Africa, and their use has well-documented consequences of co-selection of resistance toward other antibiotics, which may lead to co-transmission of multiple antibiotic resistance genes [67,70]. The use of olaquindox as a growth promotor should be reconsidered in the South African context, taking food security, economic productivity, animal welfare and the emergence of antibiotic resistance into account. Plasmid-mediated resistance toward colistin was not detected in human or porcine *E. coli* isolates collected from this farm. This may be linked to the ban on colistin use in livestock by the South African Veterinary Council after detection of the *mcr-*1 gene in South Africa in 2015 by Perreten and colleagues (2016) in animals and by Coetzee and colleagues (2016) in humans [72,73,74].

A study by Founou and colleagues (2022) investigated five extended-spectrum β-lactams-producing (ESBL) porcine *E. coli* isolates obtained from two abattoirs in South Africa [75]. Isolates from the same abattoir were closely related and harbored the *bla*_CTX-M-1_ and 15 genes, together with multiple other resistance determinants for fluoroquinolones, aminoglycosides, tetracyclines and trimethoprim-sulfametaoxazole [75]. In this study, ESBL *E. coli* isolates were not detected, potentially because the isolates were grown on non-selective media. It is well known that media supplemented with antibiotics lead to different isolation rates compared to non-selective media [4]. Some authors state that the use of non-selective media may underestimate the true prevalence of ESBL producers [76,77], whereas others state that it is important to monitor both dominant (non-selective media) and subdominant (selective media) microflora to accurately define the true extent of antibiotic resistance rates [78]. In future, media used for the isolation of bacteria in antibiotic resistance monitoring should be standardized to enhance the comparability of antibiotic resistance rates.

The majority of human and porcine *E. coli* isolates were non-pathogenic in this study, but different pathovars, namely EAEC, EPEC, ETEC, ExPEC and STEC, were detected at low frequencies. Enteroaggregative *E. coli* was only detected in humans, whereas EPEC, ETEC and STEC were predominantly isolated from pigs. Overlaps of pathovar-specific genes were also observed, as evidenced by the detection of the *ast*A gene, which encodes the enteroaggregative *E. coli* heat-stable enterotoxin (EAST1). This gene has previously been associated with EAEC in humans, but it is also reported as an important virulence factor of ETEC in piglets [48,79]. In this study, the *ast*A gene was also detected in the porcine EPEC isolates, as well as human EAEC isolates. In addition, the *ast*A gene was more frequently detected in porcine *E. coli* isolates than in human *E. coli* isolates. This suggests that the *ast*A gene may play a different role in disease based on the host species.

Sequence type complex 10 was the most dominant STc in both human and porcine *E. coli* isolates based on Achtman’s 7-gene MLST scheme. A study by Peng and colleagues (2022) sequenced 1871 *E. coli* isolates obtained from pigs and their immediate environment from 31 provinces in China and compared these genomes with publicly available human *E. coli* genomes [80]. The authors also found STc10 to be the most predominant ST, as in this study.

Core-genome MLST and HC showed that human and porcine *E. coli* isolates were overall genetically diverse in this study, but there was some evidence (albeit very little) of HC of isolates at levels HC:2–HC:200. This indicates (i) the transmission between pigs from different production houses, phases and sites; (ii) the transmission between pigs and humans, potentially due to proximity; and (iii) the transmission between humans, potentially due to shared facilities [13]. These findings are in line with a previous study by Muloi and colleagues (2022) in Nairobi, Kenya, which showed that the transmission of *E. coli* between humans and animals can occur, but it remains an infrequent event [81]. The findings are also in agreement with a longitudinal study conducted by Dohmen and colleagues (2023) on Dutch pigs and farm workers over six months [82]. Dohmen and colleagues found evidence of antibiotic resistance gene transmission among workers and pigs, either vertically or horizontally, in 11 of the 39 pig farms studied [82]. More transmission events could have potentially occurred on the farm in this study, but antibiotic resistance transmission routes based on plasmid type through the food chain and the environment were not investigated.

The study has some limitations, as only a single pig farm was included, which has implications for the generalizability of the findings. African swine fever was in circulation in South Africa at the time of sample collection, and multiple farms could therefore not be visited due to the fear of spreading disease. In addition, shortly after the first farm visit, the COVID-19 pandemic emerged. Although only a single recent transmission event between a human and porcine *E. coli* isolate was detected on this farm, it remains important to strengthen the antibiotic stewardship practices and reduce antibiotic use for growth promotion and metaphylaxis to mitigate the risk of transmission and spread of resistance linked to antibiotic usage. In future, this study can serve as a blueprint for implementing One Health antibiotic resistance surveillance programs in South Africa on a broader scale.

## Figures and Tables

**Table 1:** Phenotypic antibiotic resistance rates of human and porcine *E. coli* isolates.

Antibiotic Class	R MIC Breakpoint (µg/mL)	Human % (*n* = 63)	Pigs % (*n* =106)	Total % (*n* = 169)	*p*-Value
**Penicillins**					
Ampicillin	≥32	33.33 (21)	94.34 (100)	71.60 (121)	<0.05
Piperacillin	≥128	31.75 (20)	84.91 (90)	65.09 (110)	<0.05
**ß-lactam combination agents**					
Amoxicillin-clavulanate	≥32/16	0.0 (0)	0.0 (0)	0.0 (0)	NS
Ampicillin-sulbactam	≥32/16	9.52 (6)	40.57 (43)	28.99 (49)	<0.05
Piperacillin-tazobactam	≥128/4	0.0 (0)	0.00 (0)	0.0 (0)	NS
**Cephems**					
Cefepime	≥16	1.59 (1)	0.0 (0)	0.59 (1)	NS
Cefotaxime	≥4	0.0 (0)	0.0 (0)	0.0 (0)	NS
Cefotaxime-clavulante	>0.5	0.0 (0)	0.0 (0)	0.0 (0)	NS
Cefoxitin	≥32	0.0 (0)	0.0 (0)	0.0 (0)	NS
Ceftazidime	≥16	1.59 (1)	0.0 (0)	0.59 (1)	NS
Ceftazidime-clavulante	>0.25	0.0 (0)	0.0 (0)	0.0 (0)	NS
Cefuroxime	≥32	1.59 (1)	0.0 (0)	0.59 (1)	NS
**Monobactams**					
Aztreonam	≥16	1.59 (1)	0.0 (0)	0.59 (1)	NS
**Carbapenems**					
Doripenem	≥4	0.0 (0)	0.0 (0)	0.0 (0)	NS
Ertapenem	≥2	0.0 (0)	0.0 (0)	0.0 (0)	NS
Imipenem	≥4	0.0 (0)	0.0 (0)	0.0 (0)	NS
Meropenem	≥4	0.0 (0)	0.0 (0)	0.0 (0)	NS
**Aminoglycosides**					
Amikacin	≥64	0.0 (0)	0.0 (0)	0.0 (0)	NS
Gentamicin	≥16	0.0 (0)	6.6 (7)	4.14 (7)	NS
Streptomycin #	≥32	22.22 (14)	28.3 (30)	26.04 (44)	NS
Tobramycin	≥16	0.0 (0)	3.77 (4)	2.37 (4)	NS
**Tetracyclines**					
Tetracycline	≥16	30.16 (19)	95.28 (101)	71.01 (120)	<0.05
**Quinolones**					
Ciprofloxacin *	≥1	1.59 (1)	16.04 (17)	10.65 (18)	<0.05
Levofloxacin	≥2	0.0 (0)	4.72 (5)	2.96 (5)	NS
**Other**					
Chloramphenicol	≥32	4.76 (3)	35.85 (38)	24.26 (41)	<0.05
Colistin #$	>2	0.0 (0)	0.0 (0)	0.0 (0)	NS
Fosfomycin	≥256	0.0 (0)	1.89 (2)	1.18 (2)	NS
Tigecycline #	0.5	0.0 (0)	0.94 (1)	0.59 (1)	NS
Trimethoprim-sulfamethoxazole	≥4/76	39.68 (25)	28.3 (30)	32.54 (55)	NS

MIC = Minimum inhibitory concentration; R = Resistant; # Epidemiological cut-off values (ECOFF) reported and interpreted according to EUCAST guidelines (2023) NARMS; * As determined by the commercial broth microdilution method; $ MIC for colistin was determined using Sensititre. NS = Not statistically significant.

**Table 2:** Antibiotic resistance genes detected in human and porcine *E. coli* isolates using ResFinder (v.4.1).

Antibiotic Resistance Gene Class	Human % (*n* = 63)	Pigs % (*n* =106)	Total % (*n* = 169)	*p*-Value
**Aminoglycoside-modifying enzymes**				
** Acetyltransferases**				
ACC(3)-IId	0.0 (0)	0.94 (1)	0.59 (1)	NS
ACC(3)-IId; ACC-Ib-cr	0.0 (0)	5.66 (6)	3.55 (6)	NS
Not detected	100.00 (63)	61.11 (99)	95.86 (162)	NS
** Nucleotidyltransferases**				
ANT(3″)-Ia	19.05 (12)	50.0 (53)	38.46 (65)	<0.05
Not detected	80.95 (51)	50.0 (53)	61.54 (104)	<0.05
** Phosphotransferases**				
APH(3′)-Ia	0.0 (0)	12.26 (13)	7.69 (13)	<0.05
APH(3″)-Ib; APH(6)-Id	30.16 (19)	22.64 (24)	25.44 (43)	NS
APH(3′)-Ia; APH(3″)-Ib; APH(6)-Id	0.0 (0)	11.32 (12)	7.10 (12)	<0.05
Not detected	69.84 (44)	53.77 (57)	59.76 (101)	NS
**ß-lactam resistance genes**				
*bla*_TEM-1A_	1.59 (1)	2.83 (3)	2.37 (4)	NS
*bla*_TEM-1B_	28.57 (18)	76.42 (81)	58.58 (99)	<0.05
*bla*_TEM-1C_	0.0 (0)	2.83 (3)	1.78 (3)	NS
**ß-lactam resistance genes**				
*bla*_TEM-1B_ and *bla*_OXA-1_	0.0 (0)	5.66 (6)	3.55 (6)	NS
Multiple *bla*_TEM_ variants	1.59 (1)	1.89 (2)	1.78 (3)	NS
Not detected	68.25 (43)	10.38 (11)	31.95 (54)	<0.05
**Colistin**				
*pmr*B(V161G)	0.0 (0)	0.94 (1)	0.59 (1)	NS
**Macrolides**				
*mdf*A	95.24 (60)	97.17 (103)	96.45 (163)	NS
*mdf*A, *mph*A	4.76 (3)	2.83 (6)	3.55 (6)	NS
**Phenicols**				
*cat*A1	0.00 (0)	1.89 (2)	1.18 (2)	NS
*cml*A1	1.59 (1)	4.72 (5)	3.55 (6)	NS
*cml*A1, *cat*B3	0.0 (0)	0.94 (1)	0.59 (1)	NS
*cml*A1, *cat*B4, *flo*R	0.0 (0)	4.72 (5)	2.96 (5)	NS
*flo*R	0.0 (0)	1.89 (2)	1.18 (2)	NS
Not detected	98.41 (62)	85.85 (91)	90.53 (153)	<0.05
**Quinolones**				
** Chromosomal mutations**				
*gyr*A (S83A)	4.76 (3)	5.66 (6)	5.33 (9)	NS
*gyr*A (S83L)	6.35 (4)	23.58 (25)	17.16 (29)	<0.05
*par*C (A56T)	1.59 (1)	2.83 (3)	2.37 (4)	NS
*par*C (S80I)	1.59 (1)	0.94 (1)	1.18 (2)	NS
*par*E (I355T)	1.59 (1)	0.94 (1)	1.18 (2)	NS
** Plasmid-mediated**				
OqxAB	3.17 (2)	32.08 (34)	21.30 (36)	<0.05
OqxAB, acc-(6′)-Ib-cr, *qnr*S2	0.0 (0)	4.72 (5)	2.96 (5)	NS
OqxAB, *qnr*S1	0.0 (0)	6.60 (7)	4.14 (7)	NS
OqxAB, *qnr*S2	0.0 (0)	0.94 (1)	0.59 (1)	NS
*qnr*S1	7.94 (5)	6.60 (7)	7.10 (12)	NS
Not detected	88.89 (56)	49.06 (52)	63.91 (108)	<0.05
**Rifampicin**				
*arr*-3	0.0 (0)	5.66 (6)	3.55 (6)	NS
Not detected	100.00 (63)	94.34 (100)	96.45 (163)	NS
**Sulphonamides**				
*sul*1	4.76 (3)	12.26 (13)	9.47 (16)	NS
*sul*1, *sul*2	11.11 (7)	3.77 (4)	6.51 (11)	NS
*sul*1, *sul*3	0.0 (0)	0.94 (1)	0.59 (1)	NS
*sul*2	22.22 (14)	10.38 (11)	14.79 (25)	NS
*sul*2, *sul*3	0.0 (0)	1.89 (2)	1.18 (2)	NS
*su*l3	1.59 (1)	10.38 (11)	7.10 (12)	NS
Not detected	60.32 (38)	60.38 (64)	60.36 (102)	NS
**Tetracyclines**				
*tet*A	15.87 (10)	43.40 (46)	33.14 (56)	<0.05
*tet*A, *tet*M	0.0 (0)	4.72 (5)	2.96 (5)	NS
*tet*B	12.70 (8)	34.91 (37)	26.63 (45)	<0.05
*tet*B, *tet*M	0.0 (0)	11.32 (12)	7.10 (12)	<0.05
*tet*A, *tet*B	0.0 (0)	0.94 (1)	0.59 (1)	NS
Not detected	71.43 (45)	4.72 (5)	29.59 (50)	<0.05
**Trimethoprim**				
*drf*A1	9.52 (6)	2.83 (3)	5.33 (9)	NS
*drf*A1, *drf*A14	1.59 (1)	0.0 (0)	0.59 (1)	NS
*drf*A12	3.17 (2)	25.47 (27)	17.16 (29)	<0.05
*drf*A12, *drf*A21	0.0 (0)	3.77 (4)	2.37 (4)	NS
*drf*A14	12.70 (8)	4.72 (5)	7.69 (13)	NS
*drf*A17	4.76 (3)	2.83 (3)	3.55 (6)	NS
*drf*A21	0.0 (0)	0.94 (1)	0.59 (1)	NS
*drf*A5	1.59 (1)	0.0 (0)	0.59 (1)	NS
*drf*A7	4.76 (3)	0.0 (0)	1.78 (3)	NS
*drf*A7, *drf*A14	1.59 (1)	0.0 (0)	0.59 (1)	NS
Not detected	60.32 (38)	59.43 (63)	59.76 (101)	NS

NS = Not statistically significant.

**Table 3:** Virulence factors grouped according to pathotype in human and porcine *E. coli* isolates.

Virulence Gene Combinations	Human % (*n* = 63)	Pigs % (*n* =106)	Total % (*n* = 169)	*p*-Value
**Enteroaggregative *E. coli* (EAEC)**				
Dispersin (*aap*)	12.70 (8)	0.0 (0)	4.73 (8)	<0.05
Dispersin transporter protein (*aat*A)	9.52 (6)	0.0 (0)	3.55 (6)	<0.05
*aai*C, ORF4 and ORF4	4.76 (3)	0.0 (0)	1.78 (3)	NS
*aai*C	0.0 (0)	0.94 (1)	0.59 (1)	NS
Biogenesis of AFA-III				
*afa*ABCDE	1.59 (1)	0.0 (0)	0.59 (1)	NS
*afa*ABCDE8	1.59 (1)	0.0 (0)	0.59 (1)	NS
*afa*D	1.59 (1)	0.0 (0)	0.59 (1)	NS
Biogenesis of AAF-I				
*agg*ACD	3.17 (2)	0.0 (0)	1.18 (2)	NS
*agg*ABCD	1.59 (1)	0.0 (0)	0.59 (1)	NS
AggR transcriptional activation				
*agg*R and *aar*	4.76 (3)	0.0 (0)	1.78 (3)	NS
Other genes associated with EAEC				
*air*	7.94 (5)	0.94 (1)	3.55 (6)	NS
*pet*	1.59 (1)	0.0 (0)	0.59 (1)	NS
*pic*	4.76 (3)	0.0 (0)	1.78 (3)	NS
*sat*	15.87 (10)	0.0 (0)	5.92 (10)	<0.05
*sig*A	14.29 (9)	0.0 (0)	5.33 (9)	<0.05
**Enteropathogenic *E. coli* (EPEC)**				
Genes harbored on LEE pathogenicity island				
*eae*	1.59 (1)	3.77 (4)	2.96 (5)	NS
*esp*B	0.0 (0)	1.89 (2)	1.18 (2)	NS
*esp*A	1.59 (1)	3.77 (4)	2.96 (5)	NS
*esp*F	1.59 (1)	0.0 (0)	0.59 (1)	NS
*tcc*P	0.00 (0)	0.94 (1)	0.59 (1)	NS
*tir*	1.59 (1)	3.77 (4)	2.96 (5)	NS
Non-LEE effectors				
*cif*	1.59 (1)	3.77 (4)	2.96 (5)	NS
*esp*J	1.59 (1)	3.77 (4)	2.96 (5)	NS
*nle*A	1.59 (1)	3.77 (4)	2.96 (5)	NS
*nle*B	1.59 (1)	3.77 (4)	2.96 (5)	NS
**Enterotoxigenic *E. coli* (ETEC)**				
*ast*A	11.11 (7)	17.92 (19)	15.38 (26)	NS
F18 fimbriae (*fed*AF)	0.0 (0)	0.94 (1)	0.59 (1)	NS
*ltc*A	1.59 (1)	0.0 (0)	0.59 (1)	NS
*mcb*A	1.59 (1)	0.94 (1)	1.18 (2)	NS
STb toxin (*stb*)	0.00 (0)	3.77 (4)	2.37 (4)	NS
**Extraintestinal *E. coli* (ExPEC)**				
Group II capsule				
Only *kps*E	1.59 (1)	1.89 (2)	1.78 (3)	NS
*kps*M	1.59 (1)	0.0 (0)	0.59 (1)	NS
*kps*MII	1.59 (1)	3.77 (4)	2.96 (5)	NS
K1	7.94 (5)	0.0 (0)	2.96 (5)	NS
K5	7.94 (5)	0.0 (0)	2.96 (5)	NS
K23	1.59 (1)	0.0 (0)	0.59 (1)	NS
K52	4.76 (3)	0.0 (0)	1.78 (3)	NS
*neu*C	15.87 (10)	0.0 (0)	5.92 (10)	<0.05
Group III capsule				
K96	1.59 (1)	0.0 (0)	0.59 (1)	NS
K98	1.59 (1)	0.0 (0)	0.59 (1)	NS
**Shiga-toxin-producing *E. coli* (STEC)**				
Stx-2 (*stx*2AB)	0.00 (0)	1.89 (2)	1.18 (2)	NS

AFA = Afimbrial adhesion; AAF = Aggregative adherence fimbria; LEE = Locus of enterocyte effacement pathogenicity island. NS = Not statistically significant.

**Table 4:** Phylogroups in human and porcine *E. coli* isolates according to Clermont typing by EnteroBase.

Phylogroups	Human	Pigs	Total	*p-*Value
	% (*n* = 63)	% (*n* = 106)	% (*n* =169)	
A	46.03 (29)	73.58 (78)	63.31 (107)	<0.05
B1	31.75 (20)	17.92 (19)	23.08 (39)	NS
B2	4.76 (3)	0.0 (0)	1.78 (3)	NS
C	0.0 (0)	0.94 (1)	0.59 (1)	NS
Cryptic	1.59 (1)	0.94 (1)	1.18 (2)	NS
D	11.11 (7)	1.89 (2)	5.33 (9)	<0.05
E	1.59 (1)	1.89 (2)	1.78 (3)	NS
F	1.59 (1)	0.94 (1)	1.18 (2)	NS
U/cryptic	1.59 (1)	1.89 (2)	1.78 (3)	NS

NS = Not statistically significant.

**Table 5:** Sequence type complexes in human and porcine *E. coli* isolates according to MLST.

STc *	Human % (*n* = 63)	Pigs % (*n* = 106)	Total % (*n* = 169)	*p-*Value
10 *	30.16 (19)	37.74 (40)	34.91 (59)	NS
23 (ST23)	0.0 (0)	0.94 (1)	0.59 (1)	NS
32 (ST137)	0.0 (0)	1.89 (2)	1.18 (2)	NS
69 (ST69)	6.35 (4)	0.0 (0)	2.37 (4)	<0.05
86 (ST453; ST641; ST877) *	6.35 (4)	6.60 (7)	6.51 (11)	NS
95 (ST95; ST12411) *	3.17 (2)	0.0 (0)	1.18 (2)	NS
101 (ST101)	1.59 (1)	0.0 (0)	0.59 (1)	NS
155 (ST155)	3.17 (2)	0.0 (0)	1.18 (2)	NS
156 (ST12350)	1.59 (1)	0.0 (0)	0.59 (1)	NS
165 (ST165; ST1114; ST1178; ST5455)	0.0 (0)	6.60 (7)	4.14 (7)	NS
168 (ST93; ST484)	1.59 (1)	2.83 (3)	2.37 (4)	NS
206 (ST793; ST4995) *	1.59 (1)	0.94 (1)	1.18 (2)	NS
278 (ST336; ST795) *	1.59 (1)	1.89 (2)	1.78 (3)	NS
394 (ST394)	1.59 (1)	0.0 (0)	0.59 (1)	NS
399 (ST399)	3.17 (2)	0.0 (0)	1.18 (2)	NS
467 (ST480; ST2325) *	0.0 (0)	2.83 (3)	1.78 (3)	NS
469 (ST162)	1.59 (1)	0.94 (1)	1.18 (2)	NS
522 (ST3075)	1.59 (1)	0.0 (0)	0.59 (1)	NS
Complex not assigned *	34.92 (22)	36.79 (39)	36.09 (61)	NS

* STc = Sequence type complex. ST = Sequence type. The individual STs constituting the STc are indicated in brackets if no more than four STs were detected. The distribution of the individual STs within each STc is shown in the Supplementary Material if more than four different STs were detected in a complex (Supplementary Table S2). NS = Not statistically significant.

## Data Availability

Sequencing data are available at the NCBI’s GenBank under Bioproject number: PRJNA994298 (https://www.ncbi.nlm.nih.gov/sra/PRJNA994298). Additional data generated during this study were placed in an online repository (https://doi.org/10.6084/m9.figshare.23677266.v2). The accession numbers are listed in the Supplementary Material, together with the isolate’s unique identifier.

## Supplementary Materials

The following supporting information can be downloaded at: https://www.mdpi.com/article/10.3390/antibiotics13060543/s1, Table S1: Other virulence genes detected in human and porcine *E. coli* isolates; Table S2: Sequence type complex composition with the different STs detected in human and porcine *E. coli* isolates.
