# Supplementary material for: Whole-Genome Sequencing of Human and Porcine Escherichia coli Isolates on a Commercial Pig Farm in South Africa
Source: Antibiotics (Basel). 2024 Jun 11;13(6):543. doi: 10.3390/antibiotics13060543 (PMC11200671; doi:10.3390/antibiotics13060543)
Supplement: Supplementary file 1 [file antibiotics-13-00543-s001.zip › antibiotics-3031649-supplementary.pdf]

antibiotics MDPI

Table S1: Other virulence genes detected in human and porcine *E. coli* isolates

Virulence factors	Humans % (n=63)	Pigs % (n =106)	Total % (n = 169)
**Adherence and colonization**			
*cap*U	4.76 (3)	0.94 (1)	2.37 (4)
*eil*A	12.70 (8)	1.89 (2)	5.92 (10)
*etp*D	1.59 (1)	0.00 (0)	0.59 (1)
*ets*C	6.35 (4)	2.83 (3)	4.14 (7)
gad	49.21 (31)	31.13 (33)	37.87 (64)
hra	14.29 (9)	26.42 (28)	21.89 (37)
*ibe*A	1.59 (1)	0.00 (0)	0.59 (1)
iha	42.86 (27)	0.00 (0)	15.98 (27)
iss	58.73 (37)	26.42 (28)	38.46 (65)
*irp*2	38.10 (24)	12.26 (13)	21.89 (37)
*kat*P	1.59 (1)	5.66 (6)	4.14 (7)
*lpf*A	41.27 (26)	24.53 (26)	30.77 (52)
*omp*T	44.44 (28)	21.70 (23)	30.18 (51)
*pap*A_F9	3.17 (2)	0.00 (0)	1.18 (2)
*pap*A_F11	3.17 (2)	0.00 (0)	1.18 (2)
*pap*A_F19	0.00 (0)	0.94 (1)	0.59 (1)
*pap*A_F20	0.00 (0)	0.94 (1)	0.59 (1)
*pap*A_F43	3.17 (2)	0.00 (0)	1.18 (2)
*pap*A_*fei*A_F8	1.59 (1)	0.00 (0)	0.59 (1)
*pap*A_*fsi*A_F16	3.17 (2)	0.00 (0)	1.18 (2)
F17 (*f*17A; *f*17G)	1.59 (1)	0.00 (0)	0.59 (1)
*pap*C	9.52 (6)	11.32 (12)	10.65 (18)
*tra*T	60.32 (38)	41.51 (44)	48.52 (82)
tsh	3.17 (2)	13.21 (14)	9.47 (16)
usp	6.35 (4)	0.00 (0)	2.37 (4)
*yfc*V	4.76 (3)	0.00 (0)	1.78 (3)
**Colicins**			
cba	0.00 (0)	7.55 (8)	4.73 (8)
cea	11.11 (7)	3.77 (4)	6.51 (11)
celb	6.35 (4)	7.55 (8)	7.10 (12)
cia	9.52 (6)	7.55 (8)	8.28 (14)
cma	3.17 (2)	12.26 (13)	8.88 (15)
**Iron acquisition**			
*chu*A	20.63 (13)	5.66 (6)	11.24 (19)
*fyu*A	39.68 (25)	12.26 (13)	22.49 (38)
*ire*A	19.05 (12)	0.94 (1)	7.69 (13)
*iro*N	9.52 (6)	0.94 (1)	4.14 (7)
*iuc*C	41.27 (26)	0.94 (1)	15.98 (27)
*iut*A	9.52 (6)	0.94 (1)	4.14 (7)
*sit*A	36.51 (23)	5.66 (6)	17.16 (29)
**Microcins**			
*cva*B	3.17 (2)	0.00 (0)	1.18 (2)
*mch*B	4.76 (3)	5.66 (6)	5.33 (9)
*mch*C	3.17 (2)	5.66 (6)	4.73 (8)
*mch*F	7.94 (5)	6.60 (7)	7.10 (12)
*mcm*A	0.00 (0)	5.66 (6)	3.55 (6)

**Virulence factors**	**Humans % (n=63)**	**Pigs % (n =106)**	**Total % (n = 169)**
**Serine protease autotransporters (SPATE)**			
*esp*I	9.52 (6)	0.00 (0)	3.55 (6)
*esp*P	4.76 (3)	0.00 (0)	1.78 (3)
*sep*A	3.17 (2)	2.83 (3)	2.96 (5)
**Toxins**			
*hly*F	6.35 (4)	0.94 (1)	2.96 (5)
*sen*B	12.70 (8)	0.00 (0)	4.73 (8)
*tox*B	0.00 (0)	1.89 (2)	1.18 (2)
vat	3.17 (2)	0.00 (0)	1.18 (2)

Table S2: Sequence type complex composition with the different STs detected in human and porcine *E. coli* isolates

Sequence type (ST) complex	Sequence type (ST)	Humans % (n=63)	Pigs % (n =106)	Total % (n = 169)
**ST 10 complex**	10	19.05 (12)	24.53 (26)	22.49 (38)
	34	3.17 (2)	0.94 (1)	1.78 (3)
	48	0.00 (0)	1.89 (2)	1.18 (2)
	215	0.00 (0)	3.77 (4)	2.37 (4)
	218	3.17 (2)	0.94 (1)	1.78 (3)
	227	1.59 (1)	0.00 (0)	0.59 (1)
	2223	0.00 (0)	0.94 (1)	0.59 (1)
	3877	1.59 (1)	0.00 (0)	0.59 (1)
	4427	0.00 (0)	0.94 (1)	0.59 (1)
	10170	0.00 (0)	0.94 (1)	0.59 (1)
	11222	0.00 (0)	0.94 (1)	0.59 (1)
	12410	1.59 (1)	0.00 (0)	0.59 (1)
	12515	0.00 (0)	0.94 (1)	0.59 (1)
	12416	0.00 (0)	0.94 (1)	0.59 (1)
**ST complex 86**	453	3.17 (2)	0.94 (1)	1.78 (3)
	641	3.17 (2)	3.77 (4)	3.55 (6)
	877	0.00 (0)	1.89 (2)	1.18 (2)
**ST complex 95**	95	1.59 (1)	0.00 (0)	0.59 (1)
	12411	1.59 (1)	0.00 (0)	0.59 (1)
**ST complex 165**	165	0.00 (0)	0.94 (1)	0.59 (1)
	1114	0.00 (0)	3.77 (4)	2.37 (4)
	1178	0.00 (0)	0.94 (1)	0.59 (1)
	5455	0.00 (0)	0.94 (1)	0.59 (1)
**ST complex 168**	93	0.00 (0)	2.83 (3)	1.78 (3)
	484	1.59 (1)	0.00 (0)	0.59 (1)
**ST complex 206**	793	1.59 (1)	0.00 (0)	0.59 (1)
	4995	0.00 (0)	0.94 (1)	0.59 (1)
**ST complex 278**	336	1.59 (1)	0.00 (0)	0.59 (1)
	795	0.00 (0)	1.89 (2)	1.18 (2)
**ST complex 467**	480	0.00 (0)	0.94 (1)	0.59 (1)
	2325	0.00 (0)	1.89 (2)	1.18 (2)
**Complex not assigned**	-2332	0.00 (0)	0.94 (1)	0.59 (1)
	117	0.00 (0)	0.94 (1)	0.59 (1)
	154	0.00 (0)	4.72 (5)	2.96 (5)
	202	0.00 (0)	1.89 (2)	1.18 (2)
	216	3.17 (2)	0.00 (0)	1.18 (2)
	224	1.59 (1)	0.00 (0)	0.59 (1)
	362	1.59 (1)	0.00 (0)	0.59 (1)
	401	1.59 (1)	0.00 (0)	0.59 (1)
	452	1.59 (1)	0.00 (0)	0.59 (1)
	500	0.00 (0)	0.94 (1)	0.59 (1)
	542	0.00 (0)	9.43 (10)	5.92 (10)
	543	1.59 (1)	0.00 (0)	0.59 (1)
	678	1.59 (1)	0.00 (0)	0.59 (1)
	871	1.59 (1)	1.89 (2)	1.78 (3)

Sequence type (ST) complex	Sequence type (ST)	Humans % (n=63)	Pigs % (n =106)	Total % (n = 169)
**Complex not assigned**	1056	1.59 (1)	0.00 (0)	0.59 (1)
	1081	0.00 (0)	2.83 (3)	1.78 (3)
	1112	1.59 (1)	0.94 (1)	1.18 (2)
	1146	1.59 (1)	0.00 (0)	0.59 (1)
	1571	1.59 (1)	0.00 (0)	0.59 (1)
	1716	0.00 (0)	0.94 (1)	0.59 (1)
	2144	3.17 (2)	0.00 (0)	1.18 (2)
	2522	1.59 (1)	0.00 (0)	0.59 (1)
	3947	1.59 (1)	0.94 (1)	1.18 (2)
	4214	0.00 (0)	2.83 (3)	1.78 (3)
	5308	1.59 (1)	0.00 (0)	0.59 (1)
	5617	1.59 (1)	0.00 (0)	0.59 (1)
	5951	0.00 (0)	0.94 (1)	0.59 (1)
	7651	0.00 (0)	0.94 (1)	0.59 (1)
	9063	1.59 (1)	0.00 (0)	0.59 (1)
	9628	0.00 (0)	0.94 (1)	0.59 (1)
	10907	1.59 (1)	0.00 (0)	0.59 (1)
	12412	1.59 (1)	0.00 (0)	0.59 (1)
	12413	0.00 (0)	0.94 (1)	0.59 (1)
	12414	0.00 (0)	0.94 (1)	0.59 (1)
